# An Overview of Metabolic Phenotyping in Blood Pressure Research

**DOI:** 10.1007/s11906-018-0877-8

**Published:** 2018-07-10

**Authors:** Ioanna Tzoulaki, Aikaterini Iliou, Emmanuel Mikros, Paul Elliott

**Affiliations:** 1 Department of Epidemiology and Biostatistics, School of Public Health, Imperial College London, London, UK; 2 MRC-PHE Centre for Environment and Health, School of Public Health, Imperial College London, London, UK; 3 Department of Hygiene and Epidemiology, University of Ioannina Medical School, Ioannina, Greece; 4 Department of Pharmaceutical Chemistry, Faculty of Pharmacy, University of Athens, Athens, Greece; 5 Health Data Research UK (HDR-UK), London, UK; 6 Dementia Research Institute at Imperial College London, London, UK

**Keywords:** Blood pressure, Hypertension, Metabolomics, Metabolic phenotyping, Microbiome, Epidemiological studies

## Abstract

**Purpose of the Review:**

This review presents the analytical techniques, processing and analytical steps used in metabolomics phenotyping studies, as well as the main results from epidemiological studies on the associations between metabolites and high blood pressure.

**Recent Findings:**

A variety of metabolomic approaches have been applied to a range of epidemiological studies to uncover the pathophysiology of high blood pressure. Several pathways have been suggested in relation to blood pressure including the possible role of the gut microflora, inflammatory, oxidative stress, and lipid pathways. Metabolic changes have also been identified associated with blood pressure lowering effects of diets high in fruits and vegetables and low in meat intake. However, the current body of literature on metabolic profiling and blood pressure is still in its infancy, not fully consistent and requires careful interpretation.

**Summary:**

Metabolic phenotyping is a promising approach to uncover metabolic pathways associated with high blood pressure and throw light into the complex pathophysiology of hypertension.

## Introduction

High blood pressure is the leading modifiable cause of death worldwide; even small increments in blood pressure are associated with an increased risk of cardiovascular disease (CVD) [1•, 2]. Extensive research has confirmed independent unfavorable additive effects on blood pressure of adiposity (body mass), excess alcohol use, high salt intake, and inadequate potassium intake, as well as beneficial effects from use of the Diet Approaches to Stop Hypertension (DASH) feeding trial combination diet [3–8]. Yet the effect of other nutrients, environmental factors as well as the mechanisms through which known dietary factors affect blood pressure are less well understood.

Metabolic phenotyping (metabolomics), the study of low molecular weight molecules or metabolites produced within cells and biologic systems, offers a promising approach to measure biological effects of environmental and other exposures and discover novel disease biomarkers [9••] with applications to blood pressure research. Here, we outline the main analytical approaches in metabolic phenotyping research and then discuss its application to blood pressure research in human studies, focusing on recent epidemiological studies investigating metabolic markers and pathways associated with blood pressure.

## Metabolic Phenotyping

High-throughput metabolic phenotyping or profiling is a powerful tool in systems biology [10] that is being increasingly applied to biomarker discovery. It refers to the biochemical quantitative analysis of multiple metabolites in biological fluids, tissues, and tissue extracts. Specifically, global (untargeted) metabolic profiling provides a comprehensive assessment of the metabolic effects of intrinsic markers and extrinsic exposures (the internal and external exposome) from a variety of sources including, but not limited to, dietary, lifestyle, gut microbial, and psychosocial factors [9••, 11, 12•]. These factors interact at a cellular and systems level to generate a metabolic signature characteristic of health or disease [13].

**Fig. 1:**
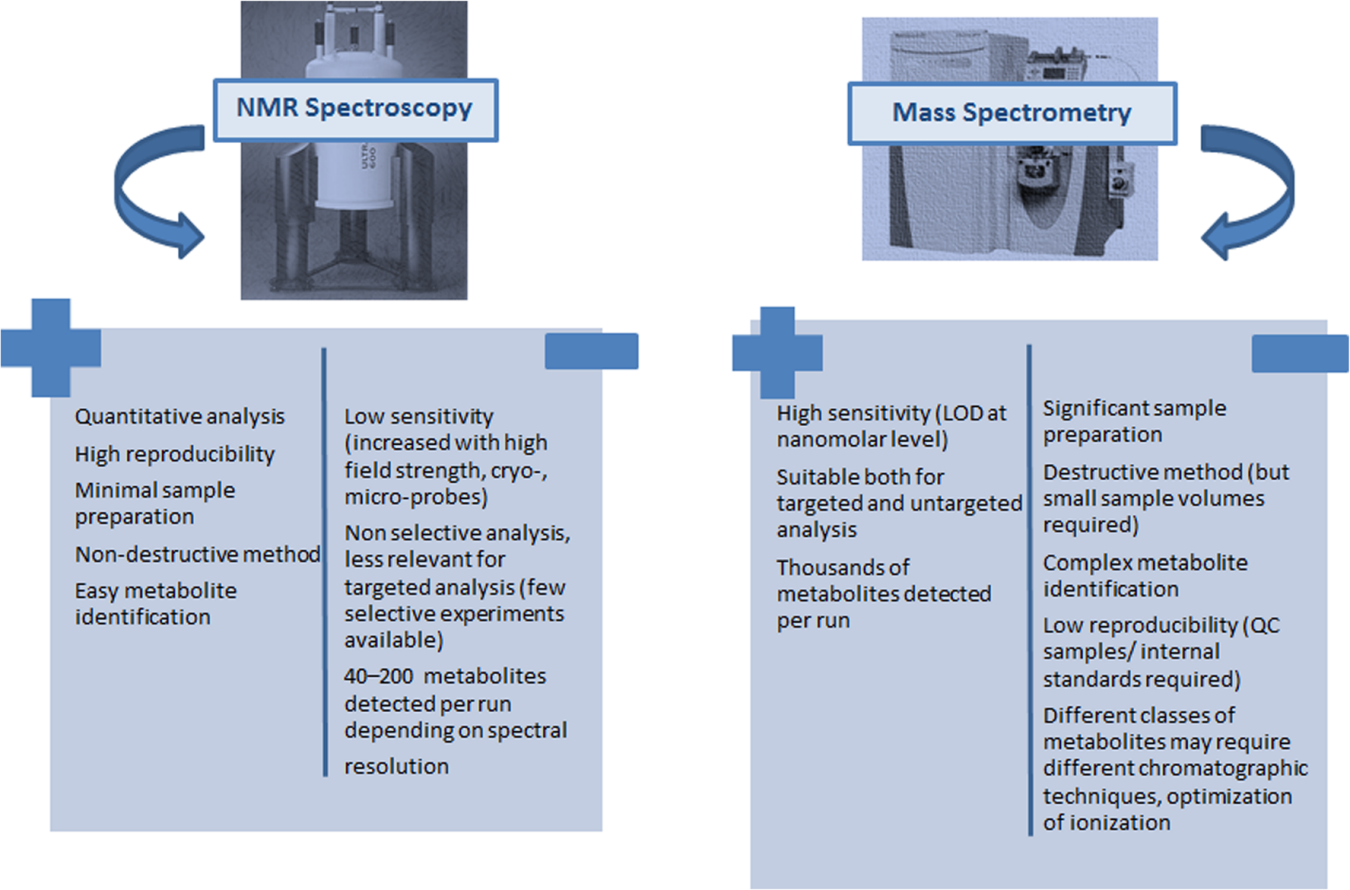
Strengths and weaknesses of NMR Spectroscopy and MS spectrometry as an analytical technique in metabolomics research

Low molecular weight metabolites are intermediates or end products of cellular processes and as an ensemble characterise the function of an organism. The main analytical methods used are proton Nuclear Magnetic Resonance (^1^H NMR) spectroscopy, and Mass Spectrometry (MS) coupled to an array of separation techniques including Gas Chromatography (GC) and Liquid Chromatography (LC). These methods are complementary to each other [14] with different strengths and weaknesses (Fig. 1) and allow the simultaneous detection and quantitation of a variety of low-molecular-weight metabolites including carbohydrates, lipids, organic acids, amino acids, energy-related metabolites and gut microbial co-metabolites, with concentrations ranging from picomoles per litre to millimoles per litre. The wealth of resulting spectral data can be analysed using emerging methods for automated data reduction and pattern recognition techniques (multivariate chemometric analysis) leading to the efficient exploitation of complex spectral profiles and extraction of latent information [15–18].

^1^H NMR spectroscopy is based on the excitation properties of protons in the presence of magnetic field. NMR-active nucleus absorbs electromagnetic radiation at characteristic frequencies providing information about the structure and abundance of the molecule, enabling both identification and quantitation. NMR is a non-destructive method characterised by high reproducibility [19–21]. Different NMR techniques can be used for particular type of analysis like magic angle spinning (MAS)-NMR to study intact tissues or lipoprotein profiling for lipoprotein particle number and size, and lipoprotein subfractions in blood samples by scrutinising the shape of the signal envelopes [22]. NMR is restricted by means of sensitivity and the number of metabolites that can be detected by a single run. High abundance metabolites may cause masking of lower abundance molecules. To overcome the latter, prior sample preparation [23, 24] (e.g. separation between aqueous and lipid phase), two-dimensional NMR spectra [25], or hyphenation of NMR with separation techniques [26] can be used.

By contrast, MS is characterised by high sensitivity (1 pmol/L), allowing the detection of less abundant metabolites. It is particularly useful for global metabolite profiling, as thousands of metabolites can be detected in a single run [27, 28]. Its principle is that molecules are charged or ionised and then charged molecules and their fragments are separated based on the *m*/*z* ratio (mass-to-charge ratio). In metabolic phenotyping, where small molecules are studied, *z* is equal to one, so that *m*/*z* ratio coincides with the mass. Different ionisation techniques can be used, with Electrospray Ionization Technique (ESI), both in negative and positive mode, being the most applicable in metabolic phenotyping research [29, 30]. Major disadvantages of MS technique include that it is a destructive method, though a smaller sample is needed compared to NMR. Attention should be paid to sample preparation, as hydrophobicity and ionisation potential affects the analysis. When hyphenated with GC, a derivatisation process is needed [31•]. Moreover, MS is characterised by low reproducibility, necessitating the use of internal standards and quality control samples [32]. The latter may be pooled samples, consisting of a small amount of each one of the study samples, predetermined mixtures of “representative” metabolites or samples commercially available.

Metabolic phenotyping through NMR or MS can be hypothesis driven, named as a targeted approach [33], where only a preselected group of metabolites is measured, usually related to the pathway of interest or a specific class of metabolites. In targeted methods, chemical identification (annotation) of the metabolites is completed at the development phase and also serves for validation of potential biomarkers previously discovered. On the other hand, untargeted approaches are hypothesis generating and allow for unbiased detection of a wide range of metabolites and therefore may help reveal novel metabolic pathway or biomarkers. However, structural identification of the metabolic features can be complex and often annotation of the metabolic features that are of interest is incomplete [27, 34, 35].

The most frequently used samples in human metabolomics studies are blood and urine as they are relatively easy to collect and provide a comprehensive overview of various system level metabolic pathways [36, 37]. Metabolic phenotyping can also be undertaken of other biological fluids (e.g. saliva), tissue extracts from biopsies, or cell extracts [38–41]. These samples may reflect pathophysiological changes in specific tissues or cells that may not be captured by blood and urine.

**Fig. 2:**
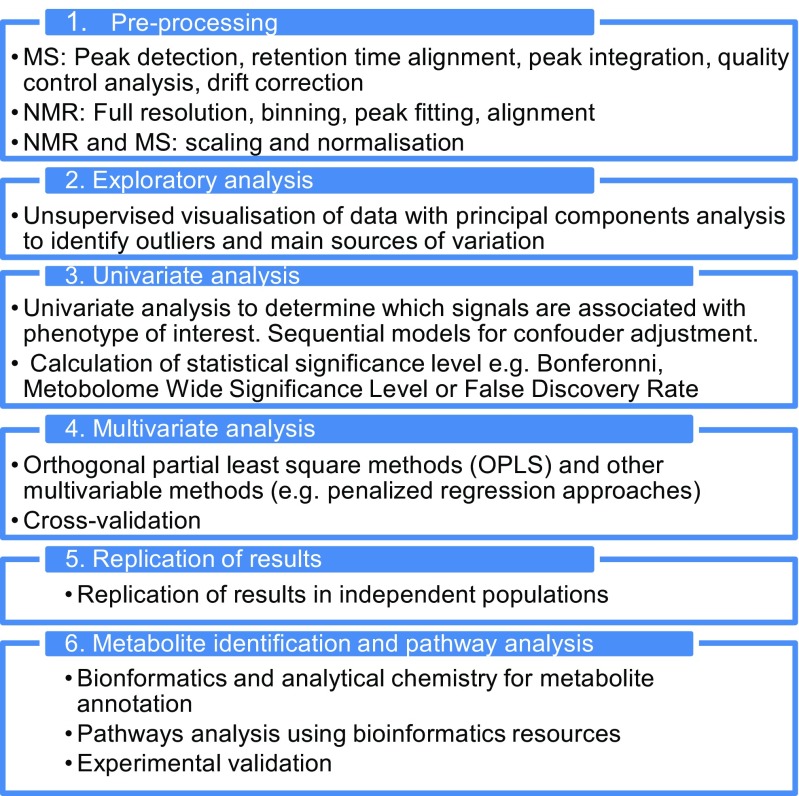
Data analysis workflow for metabolic phenotyping studies

Data analysis of metabolic phenotyping data is relatively complex (Fig. 2). It may comprise, among others, initial steps of processing to correct for instrumental drift [42, 43], methods for peak alignment/integration [44–46], correction of sample dilution effects [47], quality control of variables, and later use of multi- or univariate statistical methods adjusting for covariates, in order to find associations with health outcomes. The complexity of the task increases in studies with subsets of data acquired by different platforms, instruments, and methods [31•, 48•]. In untargeted analyses in particular, attention needs to be paid to the high dimensionality of the data, the large inter-correlations between data points, and the problems of multiple testing [31•, 49].

## Epidemiological Studies on Metabolic Phenotyping in Blood Pressure Research

Applications of metabolic phenotyping to blood pressure research have been multifaceted, including the study of lifestyle and environmental factors on blood pressure levels through biomarkers of exposure [50–61], investigation of disease pathophysiological processes [62–70] and evaluation of drug response to treatment for high blood pressure and associated side effects [71–77]. However, despite increasing interest in this area, most studies are still small, cross-sectional, and limited to targeted metabolic platforms.

By far, the largest piece of evidence regarding small metabolites and pathogenesis of high blood pressure comes from the INTERnational collaborative study on Macro/micronutrients And blood Pressure (INTERMAP) that included 4630 men and women aged 40–59 years from 17 population samples in the USA, UK, Peoples’ Republic of China, and Japan [55••]. The study has a unique collection of four interviewer-administered multi-pass 24-h dietary recalls allowing for a comprehensive assessment of dietary habits, and two 24-h urine collections from each individual. ^1^H NMR-based untargeted metabolic phenotyping of urine was performed on the stored 24-h urine collections from the 4630 participants. These analyses highlighted discriminatory metabolites across the four countries, of which four metabolites (alanine, hippurate, formate, and *N*-methylnicotinate) were associated with blood pressure of individuals. Two of these metabolites were inversely related to blood pressure, formate (a byproduct of fermentation of dietary fibre by the gut microbiome), and hippurate (formed by hepatic glycine conjugation of benzoate, derived from gut microbial fermentation of plant phenolics). Alanine, on the other hand, which is higher in people who predominantly consume animal rather than vegetable products, was directly associated with blood pressure. Hippurate and 2-hydroxy-isobutyrate, derived from microbial degradation of dietary proteins, also discriminated between North and South Chinese populations, at differing cardiovascular risk (higher blood pressure and rates of stroke in North vs South China) [60].

The importance of the gut microbiome in cardiovascular phenotypes such as blood pressure is further supported by studies showing a link between trimethylamine-*N*-oxide (TMAO) and atherosclerosis [78]; TMAO, a small-molecule metabolite derived from the metabolism of dietary phosphatidylcholine, has been associated with risk of atherosclerosis and heart disease in observational studies and mouse models [79, 80]. In relation to blood pressure, Li et al. found decreased microbial richness and diversity in prehypertensive and hypertensive populations compared with healthy controls [81•] while in rats, TMAO infusion was found to enhance the hypertensive effects of angiotensin II [82].

A role for the gut microbiome in high blood pressure was also reported in analysis of data from 896 normotensive black participants in the Atherosclerosis Risk in Communities (ARIC) study [57]. Serum samples were analysed by GS-MS using the Metabolon platform; 4-hydroxyhippurate, an end product of benzoate metabolism from microbial fermentation of polyphenols, was associated with 17% higher risk of hypertension at 10 years follow-up, after adjusting for baseline blood pressure and traditional risk factors (hazard ratio per SD (95%CI) 1.17 (1.08, 1.28)). In addition, a sex steroid pattern derived from principal components analysis (PCA) was positively associated with elevated risk of incident hypertension (highest versus lowest quintile of the sex steroid scores, HR 1.72).

In the European Prospective Investigation into Cancer and Nutrition (EPIC)-Postdam study [68••], 127 metabolites were analysed among 135 cases (participants who developed hypertension over 10 years follow-up) and 981 non-cases, using a targeted MS platform in blood samples (Biocrates); serine, glycine, acyl-alcyl-phospatidylcholines (PCs), and diacyl-PCs were associated with incident hypertension. These results suggest a possible role of inflammatory pathways in high blood pressure; both serine and glycine share anti-inflammatory and antioxidant properties, while PCs may also exhibit an anti-inflammatory role and protect lipoproteins from oxidation. Other cross-sectional studies with targeted metabolic profiling have supported the role of inflammation and oxidative stress in high blood pressure [65, 70].

Finally, a number of studies have supported the associations between lipid [63••, 66, 83] and amino acid [62, 64, 68••] metabolism with blood pressure. The TWINUK study with measurement of 280 metabolites in fasting serum samples (MS-based metabolic profiling using Metabolon platform) on 3580 females with replication in two independent cohorts: Cooperative Health Research in the Augsburg Region (KORA) (*n* = 1494) and Hertfordshire (*n* = 1515) [63••] showed direct associations between hexadecanedioate (dicarboxylic acid) and both blood pressure and all-cause mortality. A causal role of this metabolite on blood pressure was supported by in vivo studies in rats highlighting the potential role of fatty acid ω-oxidation in blood pressure regulation. Other smaller studies also suggested several lipids and amino acids associated with blood pressure levels [67, 69].

## Metabolic Phenotyping, Dietary Intervention Studies, and Blood Pressure

The response of blood pressure regulation to dietary interventions has been investigated through metabolic phenotyping approaches in several studies, where different dietary patterns were adopted including the DASH Diet [50–52], Optimal Macronutrient Intake Trial for Heart Health (OmniHeart) Diets [56••], and Mediterranean Diet [61].

The metabolic response to OmniHeart diet [56••] was studied by ^1^H NMR of stored 24-h urine samples among 58 individuals with prehypertension or stage 1 hypertension. In a randomised crossover design, participants received a carbohydrate rich (OMniCarbo), a protein rich (OmniProt), or a monounsaturated fat-rich (OmniMFA) diet, for 6 weeks each. Blood pressure was significantly associated with six urinary metabolites reflecting (i) dietary intake: proline-betaine (inverse) and carnitine (direct); (ii) gut microbial co-metabolites: hippurate (direct), 4-cresyl sulfate (inverse), and phenylacetylglutamine (inverse); and (iii) tryptophan metabolism: N-methyl-2-pyridone-5-carboxamide (inverse). These results demonstrate, in a trial setting, changes in metabolic profiles from manipulation of dietary macronutrient content that lowers blood pressure levels.

Metabolic phenotyping in relation to dietary sodium reduction has also been studied. The response of dietary sodium reduction (DSR) was studied in a 10-week crossover RCT study among 17 adults with elevated systolic blood pressure (130–159 mmHg) [51]. Ten (of 289) measured urinary metabolites were significantly altered (nine upregulated and one downregulated) during low-sodium diet. These metabolites were involved in biologic pathways of nitric oxide production, oxidative stress, and osmotic regulation. Moreover, in the DASH-sodium randomised crossover trial [52], with individuals assigned to either DASH diet or control diet for 12 weeks, receiving in random order high, medium, and low amount of sodium for 30 days, 531 plasma metabolites were measured among 73 participants at the end of their high- and low-sodium interventions and among 46 participants at the end of their high- and medium-sodium interventions. 4-Ethylphenylsulfate, a xenobiotic produced by the gut microflora related to benzoate metabolism, increased with sodium reduction, suggesting that sodium intake may affect the gut microbial activity.

## Metabolic Phenotyping and Response to Therapy

Several studies have attempted to identify potential biomarkers of drug responsiveness and to understand the molecular mechanisms that lead to drug response variation in blood pressure. For example, 313 Finnish men with high blood pressure (aged 35 to 60 years) from the Genetics of Drug Responsiveness in Essential Hypertension (GENRES) study received in a double-blind rotational design amlodipine, bisoprolol, hydrochlorothiazide (HCTZ), and losartan, each as monotherapy for 1 month, with 1-month placebo cycles between each treatment [71]. Treatment led to decreased long-chain acyl carnitines (amlodipine, bisoprolol, losartan) and medium and long-chain fatty acids (bisoprolol). Hexadecanedioate, a fatty acid previously linked to hypertension, was reduced after treatment with amlodipine, while HCTZ was, as expected, associated with increased uric acid. Although the study failed to identify a potential biomarker of drug response, it underscored the importance of fatty acids metabolism in hypertension. A lipidomics-based study [72] of 25 patients with essential hypertension on antihypertensive drug therapy, compared with 30 untreated patients with essential hypertension and 28 normotensive participants, also provided evidence supporting perturbed plasma lipid metabolism associated with hypertension. Specifically, triglycerides and total cholesteryl esters were significantly higher in hypertensive compared with normotensive participants, but significantly decreased in hypertensive patients after treatment.

## Conclusions

A variety of metabolomic approaches applied to a range of epidemiological studies has been used to uncover the pathophysiology of high blood pressure. Several pathways have been suggested in relation to blood pressure including the possible role of the gut microflora [55••, 56••, 57], inflammatory [68••], oxidative stress [65, 70], and lipid pathways [63••, 66, 83]. Metabolic changes have also been identified associated with blood pressure lowering effects of diets high in fruits and vegetables and low in meat intake [51, 52, 56••]. However, the current body of literature on metabolic profiling and blood pressure is still in its infancy, not fully consistent and requires careful interpretation. The majority of studies are small cross-sectional investigations in which the temporality of the relationship between exposure (metabolite) and outcome (high blood pressure) is uncertain, limiting any causal interpretation. In addition, independent replication efforts are rare and are further impaired by metabolic profiling methodologies that are difficult to integrate and harmonise between studies. To that end, metabolomics research should adapt practices such as those that are common in genetic epidemiology including correction for multiple testing and independent replications of findings in other populations. Similarly, functional follow-up studies that investigate the role of the metabolites in a physiological pathway such as animal models and human cell lines are necessary to further elucidate causal associations. Mendelian Randomisation approaches [84] are another approach to investigate the causality and direction of associations although they may face challenges in metabolomics research due to the large inter-correlations between metabolites and widespread pleiotropy. [85] Finally, integration of metabolomics with other omic methods is a promising approach to uncover the complex pathophysiology of hypertension by throwing light onto metabolic pathways linking, for example, genetic [86•] or epigenetic loci to high blood pressure.

### Sources of Funding

The work presented was carried out within the framework of a Stavros Niarchos Foundation grant to Aikaterini Iliou at the National and Kapodistrian University of Athens. Dr. Elliott is Director of the Medical Research Council-Public Health England Center for Environment and Health and acknowledges support from the Medical Research Council and Public Health England (MR/L01341X/1). He also acknowledges support from the National Institute of Health Research Biomedical Research Center at Imperial College London, and the National Institute of Health Research Health Protection Research Unit in Health Impact of Environmental Hazards (HPRU-2012–10141). Dr. Elliott is a UK Dementia Research Institute Professor, UK Dementia Research Institute at Imperial College London. This work was supported by the UK Dementia Research Institute which receives its funding from UK Dementia Research Institute Ltd. funded by the UK Medical Research Council, Alzheimer’s Society and Alzheimer’s Research UK. Dr. Elliott is associate director of the Health Data Research (HDR-UK) London which receives its funding from a consortium led by the UK Medical Research Council. This work used the computing resources of the UK MEDical BIOinformatics partnership (UK MED-BIO) which is supported by the Medical Research Council (MR/L01632X/1).
